# Stability of meropenem in portable elastomeric infusion devices: which protocol should be implemented in clinical practice?

**DOI:** 10.1128/spectrum.02064-23

**Published:** 2023-12-13

**Authors:** Beatriz Esteban-Cartelle, Dolores R. Serrano, Covadonga Pérez Menéndez-Conde, Noelia Vicente-Oliveros, Ana Álvarez-Díaz, Jesús Fortún Abete, Pilar Martín-Dávila

**Affiliations:** 1 Department of Pharmaceutics and Food Technology, School of Pharmacy, Complutense University of Madrid, Madrid, Spain; 2 Pharmacy Department, Ramón y Cajal Hospital, IRYCIS, Madrid, Spain; 3 Service of Infectious Diseases, Ramón y Cajal Hospital, IRYCIS, CIBERINF (Centro de Investigación Biomédica en Red de Enfermedades Infecciosas), Madrid, Insituto de Salud Carlos III, Spain; Instituto de Investigacion Sanitaria, Madrid, Spain

**Keywords:** meropenem, OPAT, outpatient parenteral antimicrobial therapy, elastomeric infusion devices, elastomers

## Abstract

Meropenem has an excellent activity against gram-positive and gram-negative bacteria, including multi-resistant microorganisms. Even though meropenem is a great candidate for outpatient parenteral antimicrobial therapy (OPAT), its physicochemical stability is a major challenge. This work aimed to demonstrate the suitability of including meropenem in OPAT by elucidating its physicochemical stability in a range of commonly prescribed concentrations within portable elastomeric infusion devices. Physical and chemical stability were evaluated at two concentrations commonly used in clinical practice (2 mg/mL and 25 mg/mL), and three temperatures (2–8°C, 25°C, and 32°C) using Accufuser portable elastomeric infusion devices. Drug adsorption onto portable elastomeric infusion devices was also determined at the end of the experiment. Meropenem stability significantly decreased at higher temperatures and when higher drug solution concentrations were used. Meropenem solutions at 2 mg/mL kept the drug content above 95% over 24 h at 2–8°C, but just for 8 h at 25°C. Nevertheless, solutions containing 25 mg/mL of meropenem showed a dramatic decrease in chemical stability after 8 h 2–8°C and just after 4 h at 25°C or 32°C. However, physical stability was kept favorable during this period. The drug adsorption on the material of the elastomeric infusion device was below 1%, indicating the suitability of the chosen device. We propose several administration protocols for meropenem in portable elastomeric infusion devices in clinical practice, according to the results obtained in our study. The results obtained in this study open up a possibility of administering meropenem in an OPAT setting despite its short stability.

**IMPORTANCE:**

Although outpatient parenteral antibiotic therapy can be a good approach to treating infections, a lack of data regarding antibiotic stability in portable elastomeric infusion devices restricts its safe and effective use. Actually, meropenem is used for prolonged periods above 24 h, and it is not physicochemically stable, which can compromise efficacy and toxicity. This work is of high importance to show the clinicians the real shelf life of meropenem when administered in portable elastomeric infusion devices. We propose several administration protocols for meropenem in portable elastomeric infusion devices in clinical practice, according to the stability drug results obtained in our study.

## INTRODUCTION

Meropenem (MEM) is a broad-spectrum carbapenem antibiotic with activity against most gram-positive and gram-negative bacteria, including multi-resistant microorganisms. It is indicated for treating numerous complicated infections, such as urinary tract infections, pneumonia, skin and soft-tissue infections. As a beta-lactam, it has a time-dependent activity, which means that the effectivity depends on the time in which the free drug plasma concentration is above the minimum inhibitory concentration (1, 2). This fact makes MEM an excellent antibiotic to be administered by continuous infusion. Nevertheless, MEM stability is a real challenge. The technical data sheet shows that MEM in clinical practice should be diluted in sodium chloride 0.9% or dextrose 5% at concentrations between 1 and 20 mg/mL. However, the solution expires after 3 h when stored at 15–25°C or 24 h at 2–8°C in sodium chloride 0.9%, while it must be used immediately after reconstitution in dextrose 5% (2).

Portable elastomeric infusion devices are sterile single-use pumps that release a selected drug with a constant flow rate. They are commonly used to perform outpatient parenteral antimicrobial therapy (OPAT). OPAT is a cost-saving method that allows administering antibiotic therapies at the patient’s home, avoiding hospitalization (3). Ideally, the antibiotic should be stable for at least 24 h, allowing a continuous infusion without the need to change the device. Nevertheless, due to instability, MEM use in OPAT is limited to a multiple-daily dosing regimen. To the best of our knowledge, there is limited information regarding the stability of MEM in portable elastomeric infusion devices. Physicochemical interactions and adsorption on the device can occur limiting even further, and hence, it is crucial to evaluate both the physical and chemical drug’s stability under different controlled conditions.

Foy et al. demonstrated a higher than 90% stability of 6 mg/mL MEM solutions at either 6 days at 6.7°C or 48 h under refrigerated conditions followed by 24 h storage at 22.5°C (4). In a different study, the stability of MEM reconstituted at 4 mg/mL was 7 days at 5°C, but it was reduced to 5 days at concentrations between 10 and 20 mg/mL (5). Akahane et al. observed instability after storage at 25°C for 24 h when the concentration was 12.5 mg/mL (6). More studies are needed with the concentrations commonly used in clinical practice. Moreover, none of these studies meet the requirements established by the Yellow Cover Document (YCD): A Standard Protocol for Deriving and Assessment of Stability—Part 1 Aseptic Preparation. According to these guidelines, it is fundamental to evaluate both the physical and chemical stability of antimicrobials in the final portable elastomeric device to elucidate the feasibility for OPAT implementation in clinical practice (7). A systematic review was recently published concluding that more studies complying YCD standards are needed (8).

This work aimed to validate a high-performance liquid chromatography (HPLC) method for MEM and to determine its stability in a range of commonly prescribed concentrations within portable elastomeric infusion devices according to YCD standards. Physical and chemical stability assays at two different concentrations (2 mg/mL and 25 mg/mL) and three different temperatures (2–8°C, 25°C, and 32°C) were performed using Accufuser portable elastomeric infusion devices.

## MATERIALS AND METHODS

### Materials

For HPLC method validation, MEM (> 99% purity) was purchased from Normon (Madrid, Spain). Commercially available MEM lyophilized trihydrate powder for injection (Aurovitas 1000 mg vial Madrid, Spain) was supplied by the Hospital Universitario Ramón y Cajal (Madrid, Spain). Sodium chloride 0.9% was purchased from Baxter (Madrid, Spain). Portable elastomeric infusion devices were Accufuser C0100L 10 mL/h 300 mL purchased from Grifols (Barcelona, Spain). Purified water was obtained through an Elix 3, Millipore purified water system (Merck, Massachusetts, USA). All other chemicals and solvents were at least of ACS reagent grade and were used without further purification.

### Methods

#### HPLC method validation

The analysis was carried out at room temperature (25°C) on a modular liquid chromatograph equipped with a Jasco LC 2000-Plus series PU-1580 pump, a Jasco AS-2050 autosampler fitted to a 100 µL sampling loop, and a Jasco UV-1575 UV-visible detector. Integration of the peaks was performed with the program Borwin 1.5. The method was conducted using a reversed-phase method. The mobile phase was composed of 30 mM potassium dihydrogen phosphate adjusted to pH 3 (Mettler Toledo MP230 GLP Research pH meter) with orthophosphoric acid and acetonitrile (85:15; vol/vol). The solution was filtered through a 0.45 µm membrane filter (Millipore) and sonicated before use. It was eluted isocratically at 1 mL/min and detected at 298 nm. The column used was a BDS Hypersil C18 250 × 4.6 mm, 5 µm, and the injection volume was 50 µL (9).

The analytical method was evaluated in terms of linearity, range, precision, DL, QL, and accuracy, according to International Conference on Harmonization guidelines for validation of analytical procedures (10). Standard solutions (1 mg/mL) were obtained by accurately weighing 10 mg of MEM reference standard and adding 10 mL of Milli-Q water into a volumetric flask.

A calibration curve was constructed with nine concentrations of the standard solution ranging from 0.39 to 100 µg/mL prepared by serial dilutions. The equation of the curve was obtained by linear regression. Precision intra-day (repeatability) was determined with two replicates from nine different concentrations. SD and CV were calculated.

The limit of detection (LOD) was expressed based on two blank samples using the following equation:


(1) $$DL=X_{b1}+3S_{b1}$$

where X_b1_ is the mean concentration of the blank, and S_b1_ is the standard deviation of the blank. The limit of quantification (LOQ) was expressed based on two blank samples using equation 2:


(2) $$QL=X_{b1}+10S_{b1}$$

To determine accuracy, three standard solutions of known concentrations were prepared at 1 µg/mL, 10 µg/mL, and 20 µg/mL. The AUC obtained was interpolated in the calibration curve. The results were expressed as the percentage of error between the experimental and the theoretical concentration.

#### Chemical stability

Commercial MEM trihydrate powder for injection was reconstituted in 20 mL of sodium chloride 0.9%. Portable elastomeric infusion devices were filled with MEM in sodium chloride 0.9% until a final concentration of 2 mg/mL and 25 mg/mL in a final volume of 240 mL. The concentrations selected correspond with the higher and the lower doses commonly employed in clinical practice. Three portable elastomeric infusion devices were filled with each concentration and were stored at 2–8°C, 25°C, and 32°C, respectively, in the refrigerator and temperature-controlled ovens. Samples were withdrawn from each device (10 mL) at 0 h, 2 h, 4 h, 8 h, 24 h, and 48 h to determine chemical stability. Each sample was diluted by duplicate before analysis. Drug concentration was determined using the validated HPLC method above described. Stability was determined by calculating the percentage of MEM recovered from each of the concentrations at each time. A 95% or above of active drug was considered chemically acceptable.

#### Physical stability

Physical stability studies (visible and subvisible particles and pH) were performed at 0 h, 8 h, 24 h, and 48 h. The presence of visible particles and color change was determined by visual inspection. Subvisible particles were measured by dynamic light scattering with a Zetatrac Ultra (Microtrac Inc, USA). Mean size (nm) was determined based on the size distribution in number. Five runs of 60 s per sample were carried out. Sterile 0.9% NaCl was used as background.

#### Adsorption study

At the end of the experiment, each device was fully empty. Deionized water (100 mL) was added twice to remove any remaining drug molecules. Then, the devices were empty and filled with 50 mL of H20:MeOH (50:50; vol/vol) and sonicated during 30 min. One sample of each device was analyzed by HPLC. The percentage of drug adsorbed to the device was calculated.

#### Data analysis

Data were analyzed using GraphPad Prism version 5.0 (GraphPad Software, California, USA). Statistically significant differences (*P* < 0.05) were calculated by ANOVA and *t*-test.

## RESULTS

### HPLC method validation

**Fig 1:**
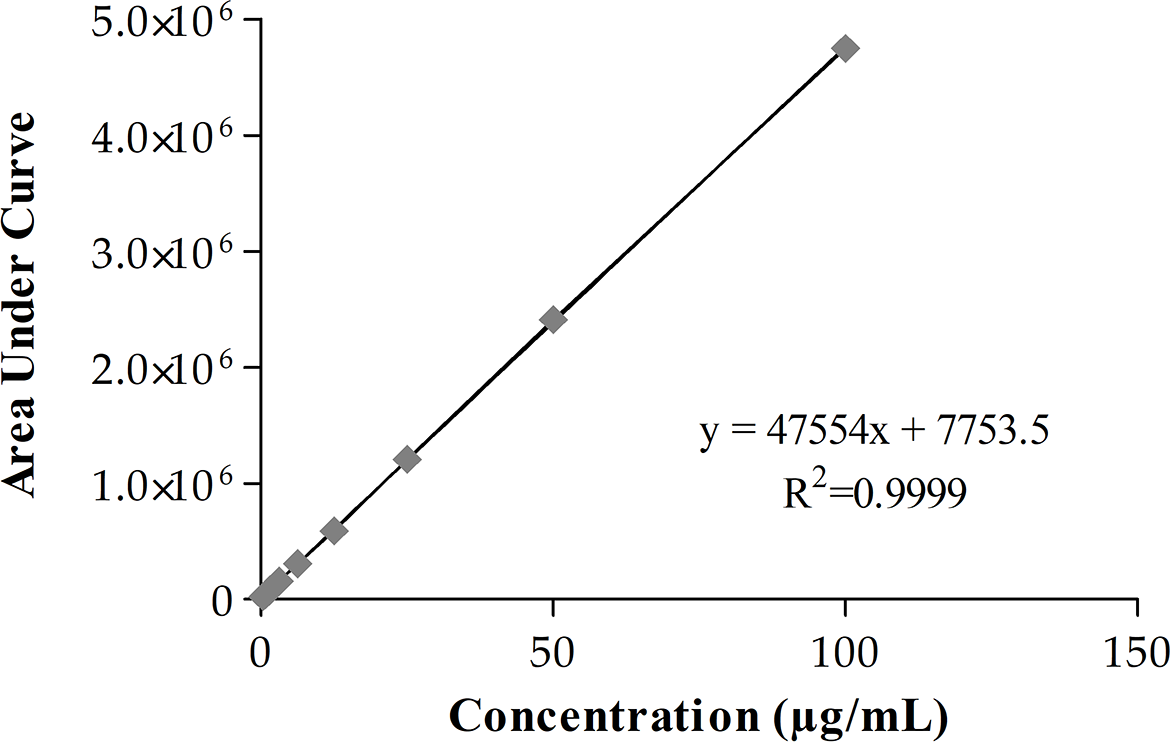
Calibration curve of MEM.

MEM showed linearity from 0.39 to 100 µg/mL range. The linear regression equation obtained was y=47554x+7753.5 with a correlation coefficient (R^2^) of 0.999. Data are shown in Fig. 1. Repeatability is shown in Table 1. MEM showed a greater coefficient of variation (CV) at higher concentrations (100 µg/mL). LOD and LOQ for MEM were 1.58 and 1.65, respectively. Accuracy data shown in Table 2. The error was below 15% at all tested concentrations.

**TABLE 1:** Repeatability and intermediate precision of MEM standards at nine different concentrations

Concn (μg/mL)	Repeatability	
	SD^*a*^, *n* = 2	CV (%), *n* = 2
100	25.53	10.62
50	11.31	8.96
25	0.07	0.12
12.5	0.85	2.88
6.25	1.34	8.53
3.125	0.57	7.16
1.5625	0.35	8.95
0.78	0.14	7.07
0.39	0.07	7.44

^*a*^ SD, standard deviation.

**TABLE 2:** Accuracy of the HPLC method for MEM

Theoretical concn (μg/mL)	Experimental concn (μg/mL)	Error (%)
20	21.69	8.46
10	11.25	12.47
1	1.11	11.07

### Chemical stability

**Fig 2:**
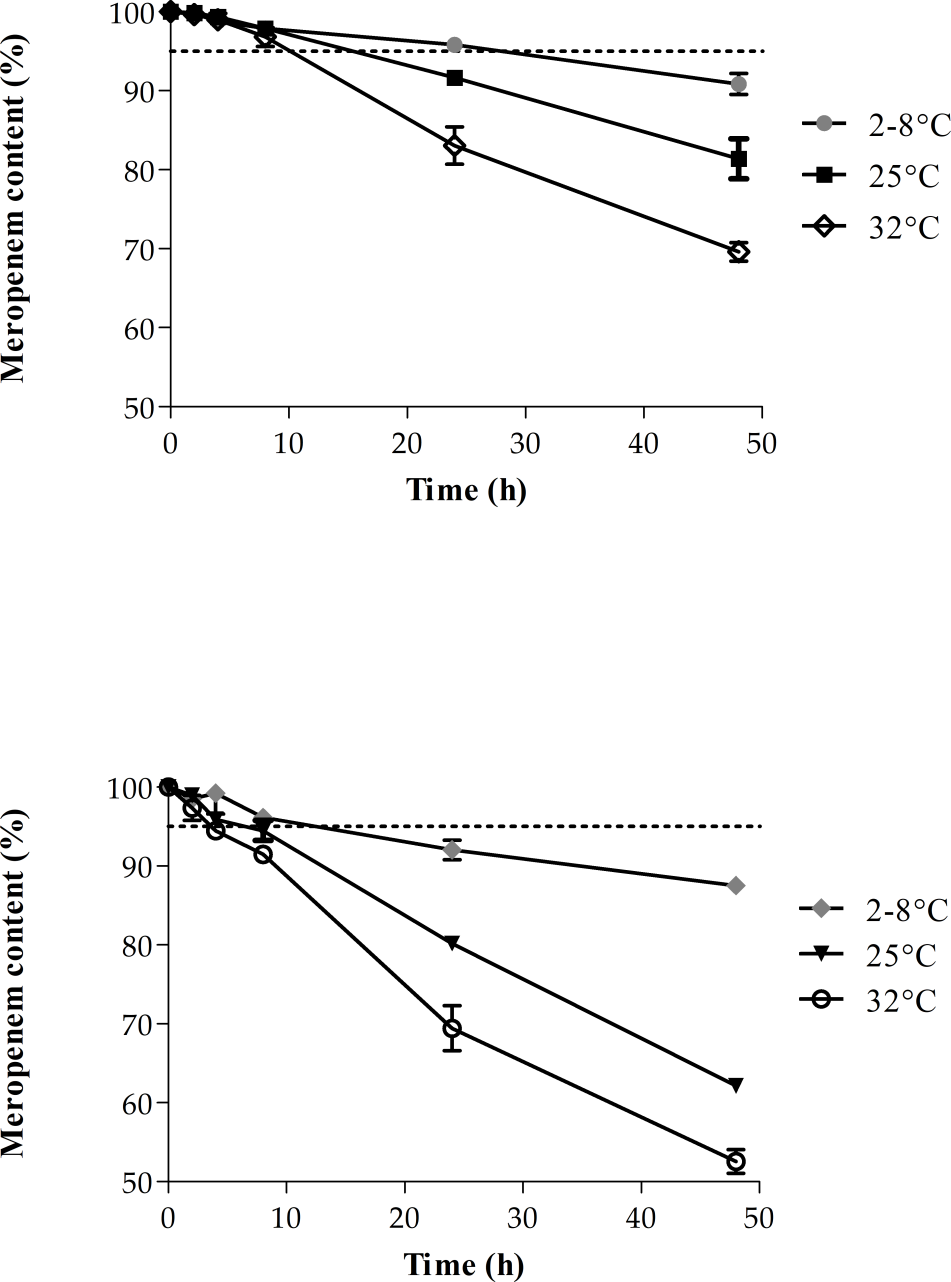
MEM content (%) remained in solution in portable elastomeric infusion devices. The stippled line marks the limit of 95% of the remaining drug which is considered an acceptable chemical stability. (**A**) Percentage of MEM remaining in 2 mg/mL solutions under refrigerated conditions, 25°C and 32°C. (**B**) Percentage of MEM remaining in 25 mg/mL solutions under refrigerated conditions, 25°C and 32°C.

The retention time for MEM was 4.6 min. The percentage of MEM remaining in the portable elastomeric infusion devices at each time and at each condition is represented in Fig. 2 and Table 3. Data were expressed as mean ± SD.

**TABLE 3:** Percentage of MEM remaining in solution in portable elastomeric infusion devices at different time points

Concn (mg/mL)	Temp (°C)	MEM remaining (% ±SD)				
		2 h	4 h	8 h	24 h	48 h
2	2–8	99.77 ± 0.51	98.83 ± 0.99	97.91 ± 0.89	95.82 ± 0.83	90.85 ± 1.32
	25	99.87 ± 0.54	99.37 ± 0.25	97.90 ± 0.40	91.63 ± 0.75	81.40 ± 2.54
	32	99.64 ± 0.47	98.46 ± 0.75	96.80 ± 1.19	83.06 ± 2.36	69.61 ± 1.15
25	2–8	98.52^*a*^	99.24 ± 2.64	96.13 ± 0.28	92.05 ± 1.23	87.50 ± 0.04
	25	98.96 ± 0.33	95.89 ± 0.39	94.50 ± 1.28	80.19 ± 0.56	62.16 ± 0.05
	32	97.36 ± 1.60	94.70 ± 0.29	91.48 ± 0.39	67.43 ± 2.87	52.56 ± 1.49

^*a*^ Due to an error with the second sample, the SD could not be calculated.

MEM was chemically stable (> 90%) for 24 h when stored under refrigerated conditions and at 25°C at the lowest concentration tested (2 mg/mL). However, according to the YCD standards, the chemical stability is recommended to be above 95%, and hence, only those solutions containing 2 mg/mL MEM in the refrigerator will be stable for 24 h. The stability was worsened at greater concentrations only remaining stable for 24 h in the refrigerator.

A statistically significant degradation was observed after 24 h in the 2 mg/mL solution (*P* = 0.0096) and after 8 h in the 25 mg/mL solutions (*P* = 0.0265) between the three temperatures tested. When comparing both concentrations, no statistically significant differences were found in the degradation of the samples stored under refrigeration after 48 h (*P* = 0.0643). On the contrary, these differences appeared after 4 h at temperatures of 25°C and 32°C (*P* = 0.0088 and *P* = 0.0156, respectively).

**Fig 3:**
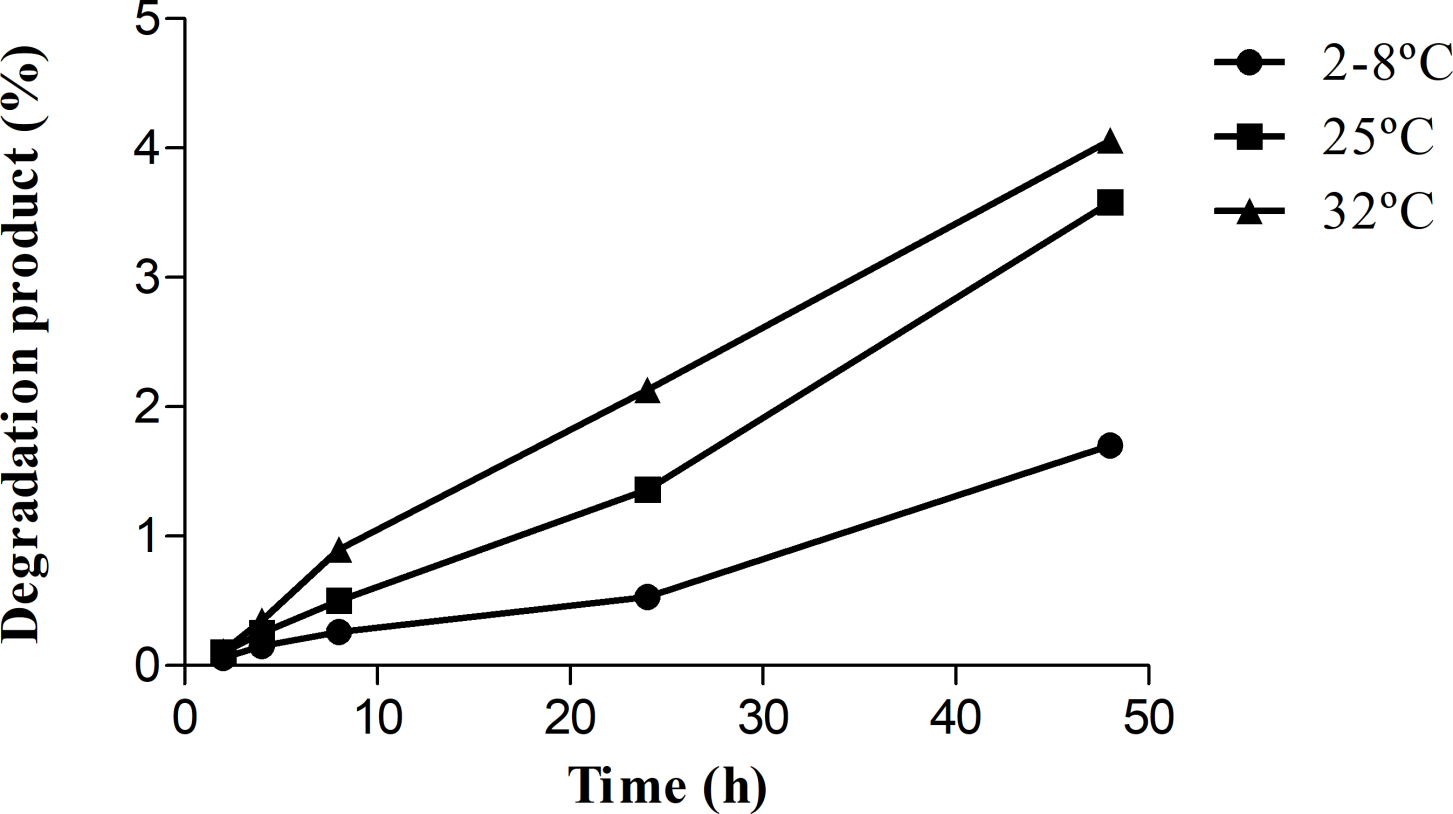
Percentage of degradation product of 25 mg/mL MEM solutions.

In 25 mg/mL solutions, a degradation product derived from MEM was observed in the chromatogram just after 2 h of storage. This degradation increased with the temperature of storage and over time. The percentage of degradation product in relation to the initial concentration of MEM is showed in Table 4 and Fig. 3. The degradation product observed in the chromatogram can be the result of a side chain modification of the MEM due to the opening of the beta-lactam ring (11).

**TABLE 4:** Percentage of degradation product in relation to the initial concentration of MEM in 25 mg/mL solutions

Concn (mg/mL)	Temp (°C)	Degradation product (%)				
		2 h	4 h	8 h	24 h	48 h
25	2–8	0.06	0.15	0.26	0.53	1.44
	25	0.10	0.25	0.50	1.36	3.58
	32	0.11	0.35	0.90	2.13	3.63

### Physical stability

#### pH modification

The pH for both concentrations of MEM solutions at the beginning of the study ranged between 7.76 and 7.96. The pH values were increased over time at all tested conditions and concentrations. However, variations were below 0.5 pH units from the starting value, which indicates stability according to the YCD limits (Table 5).

**TABLE 5:** pH values at different time points for MEM solutions at 2 mg/mL and 25 mg/mL in portable elastomeric infusion devices

Concn (mg/mL)	Temp (°C)	Time (h)			
		0	8	24	48
2	2–8	7.92	7.95	8.12	8.24
	25	7.90	7.93	8.06	8.20
	32	7.96	7.88	7.99	8.18
25	2–8	7.77	7.86	8.02	8.26
	25	7.76	7.82	7.95	8.17
	32	7.92	7.81	7.90	8.15

#### Visible and subvisible particles

Regarding visible particles, no precipitate was observed in any of the solutions throughout the study period. However, a color change was observed in 25 mg/mL solutions stored at 25°C and 32°C in which a light-yellow color appeared after 24 h. After 48 h, the color change was also visible in the solutions stored under refrigeration.

No subvisible particles were detected in any of the concentrations at all three storage conditions.

#### Adsorption study

The percentage of MEM adsorbed on the device walls was below 1% in all the conditions. Data are shown in Table 6.

**TABLE 6:** Percentage of MEM adsorbed on the portable elastomeric infusion devices after the study period

Concn (mg/mL)	Temp (°C)	Adsorbed drug (%)
2	2–8	0.03
	25	0.07
	32	0.15
25	2–8	0.004
	25	0.01
	32	0.01

#### Transfer of the results obtained to clinical practice

Table 7 proposes meropenem administration schemes in portable elastomeric infusion devices based on the results of this study. Usual recommended doses in severe infections according to renal clearance are indicated (12, 13).

**TABLE 7:** Proposed administration protocols of meropenem in clinical practice

	CrCl^*a*^ (mL/minute)	Daily dose	Recommended administration
Severe infection, including *Pseudomonas aeruginosa*	≥50	2 g every 8 h	Three 25 mg/mL portable elastomeric infusion devices over 4 h
	30–49	1 g every 8 h	Three 25 mg/mL portable elastomeric infusion devices over 4 h or three 2 mg/mL portable elastomeric infusion devices over 2 h
	10–29	1 g every 12 h	Two 25 mg/mL portable elastomeric infusion devices over 4 h or two 2 mg/mL portable elastomeric infusion devices over 2 h
Mild infection, or infections caused by non-*Pseudomonas* pathogens	>50–90	1 g every 8 h	Three 25 mg/mL portable elastomeric infusion devices over 4 h or three 2 mg/mL portable elastomeric infusion devices over 2 h
	25–50	1 g every 12 h	Two 25 mg/mL portable elastomeric infusion devices over 4 h or two 2 mg/mL portable elastomeric infusion devices over 2 h
	10–25	0,5 g every 12 h	Two 2 mg/mL portable elastomeric infusion devices over 5 h
	< 10	0,5 g every day	One 2 mg/mL portable elastomeric infusion device over 5 h

^*a*^ CrCl, creatinine clearance.

## DISCUSSION

OPAT is a novel approach implemented in hospital pharmacies over the past decade which is gaining attention due to the promotion of the quality of life of patients and the risk reduction of nosocomial infections. However, antimicrobials employed in OPAT must be stable over a certain period to allow their implementation in clinical practice. Although several stability studies of MEM have been published, to the best of our knowledge, this is the first time that the physicochemical stability is evaluated of MEM in portable elastomeric infusion devices, according to YCD standards (7). These standards, updated in 2019, set out the requirements of the stability studies for aseptic preparations.

This study evaluated the stability of MEM solutions at 2 mg/mL and 25 mg/mL in sodium chloride 0.9% in Accufuser portable elastomeric infusion devices. MEM solution at 2 mg/mL maintained above 95% of its initial drug content up to 24 h under refrigerated conditions and up to 8 h at 25°C or 32°C. The 25 mg/mL solution showed limited stability, up to 8 h at 2–8°C and just 4 h at 25°C or 32°C. During this period, physical stability in terms of pH and visible and subvisible particles was also evaluated showing no subvisible particles throughout the study. pH shifted less than 0.5 pH units of the original starting value which is considered acceptable. These data are consistent with that described by Akahane et al., who studied the stability of MEM 12.5 mg/mL solutions at 25°C and 31.1°C, finding instability within the first 8 h of the study (6). In a previous study, Smith et al. determined an MEM stability of at least 5 days of 4, 10, and 20 mg/mL solutions in sodium chloride 0.9% stored at 5°C (5). This study did not evaluate physical stability and was not following the YCD as chemical stability was considered above 90% and no 95%. However, according to our findings, MEM solutions of 25 mg/mL turned yellow after 48 h, which highlights the importance of studying physical instability. Stability of MEM solutions prepared with an adjusted 6.5 pH showed an enhanced stability over 72 h in 6 mg/mL solutions, 48 h in 12 mg/mL solutions, 24 h in 20 mg/mL solutions, and less than 24 h in 25 mg/mL solutions (4).

In this study, the stability was evaluated of the lowest and highest concentration of MEM solutions commonly used in clinical practice. Previous studies performed with cold pouches allowed keeping the temperature of the infusion elastomeric devices below 5 ˚C when replaced every 8 or 12 h (14). Based on the results obtained in our study, it could be suggested the use of ice-bricks or cold pouches around the elastomeric infusion bags to prolong the stability of the drug for at least 24 h and allow OPAT implementation, as proposed by Manning et al. (15). Nevertheless, safety and tolerability should be evaluated. Stability of MEM solutions was tested under different buffers and pH conditions. Citrate-buffered saline pH 7 (1%) improved the stability of MEM solution compared to 5% citrate buffer which was similar to saline solution 0.9% (16). Previous degradation studies investigated the degradation rate of MEM at different pH values ranging from 4.0 to 12, indicating that the pH of the highest MEM stability was between 6 and 6.5 (17). Foy et al. evaluated the stability of MEM solutions using 0.9% sodium chloride adjusted to pH 6.5 with 1 M hydrochloric acid. This strategy allowed increasing the stability of MEM solution (below 12 mg/mL) under refrigerated conditions over 3 days. However, freezing the solutions was not indicated as it triggered the formation of salt precipitates which can hamper IV administration (4). Because of these results, it would be advisable to use solutions with a pH between 6 and 6.5 to optimize the stability of meropenem. Furthermore, because meropenem is a weak acid with a pKa value of 2.9 and 7.4, these pH values would allow a more significant proportion of the drug to be in a non-ionized state to exert its pharmacological action (18).

In conclusion, stability is a key indicator for implementing antimicrobial therapy in OPAT. Ideally, the drug should maintain its physicochemical stability for at least 24 h. In this study, a validated HPLC method for MEM was developed and used. Solutions with a low concentration of MEM (2 mg/mL) could be administered over 8 h at 32°C keeping a higher than 95% drug content with no physical degradation. However, careful considerations should be taken into account when higher concentrations of MEM are used. Prolonged periods above 8 h at 2–8°C or 4 h at 25°C and 32°C are not recommended when infusing 25 mg/mL solutions. We propose several administration protocols for meropenem in portable elastomeric infusion devices in clinical practice based on the results obtained in our stability study.

The results of drug stability administered with portable elastomeric infusion devices under no-controlled conditions (real-life study) are ongoing.

## References

[1] Mattioli F , Fucile C , Del Bono V , Marini V , Parisini A , Molin A , Zuccoli ML , Milano G , Danesi R , Marchese A , Polillo M , Viscoli C , Pelosi P , Martelli A , Di Paolo A . 2016. Population pharmacokinetics and probability of target attainment of meropenem in critically ill patients. Eur J Clin Pharmacol 72:839–848. doi:10.1007/s00228-016-2053-x 27048201

[2] Meropenem Aurovit 1.000 mg polvo para solución inyectable y para perfusión EFG . 2023. Ficha Técnica Del Medicamento. Agencia Española de Medicamentos y Productos Sanitarios. Available from: https://cima.aemps.es/cima/dochtml/ft/74304/FT_74304.html

[3] Keller SC , Dzintars K , Gorski LA , Williams D , Cosgrove SE . 2018. Antimicrobial agents and catheter complications in outpatient parenteral antimicrobial therapy. Pharmacotherapy 38:476–481. doi:10.1002/phar.2099 29493791 PMC5902416

[4] Foy F , Luna G , Martinez J , Nizich Z , Seet J , Lie K , Sunderland B , Czarniak P . 2019. An investigation of the stability of meropenem in elastomeric infusion devices. Drug Des Devel Ther 13:2655–2665. doi:10.2147/DDDT.S212052 PMC6682764 31447546

[5] Smith DL , Bauer SM , Nicolau DP . 2004. Stability of meropenem in polyvinyl chloride bags and an elastomeric infusion device. Am J Health Syst Pharm 61:1682–1685. doi:10.1093/ajhp/61.16.1682 15540479

[6] Akahane M , Enoki Y , Saiki R , Hayashi Y , Hiraoka K , Honma K , Itagaki M , Gotoda M , Shinoda K , Hanyu S , Hamamura Y , Miyajima T , Ito C , Taguchi K , Uno S , Uwamino Y , Iketani O , Hasegawa N , Matsumoto K . 2021. Stability of antimicrobial agents in an elastomeric infusion pump used for outpatient parenteral antimicrobial therapy. Int J Infect Dis 103:464–468. doi:10.1016/j.ijid.2020.11.176 33246042

[7] NHS Pharmaceutical Quality Assurance Committee . 2019. A standard protocol for deriving and assessment of stability. Part 1-aseptic preparations (small molecules). 5th Ed, p 20

[8] Esteban-Cartelle B , Vicente-Oliveros N , Pérez Menéndez-Conde C , Serrano DR , Martín-Dávila P , Fortún-Abete J , León-Gil LA , Álvarez-Díaz A . 2022. Antibiotic stability in portable elastomeric infusion devices: a systematic review. Am J Health Syst Pharm 79:1355–1368. doi:10.1093/ajhp/zxac122 35511829

[9] Mendez ASL , Steppe M , Schapoval EES . 2003. Validation of HPLC and UV spectrophotometric methods for the determination of meropenem in pharmaceutical dosage form. J Pharm Biomed Anal 33:947–954. doi:10.1016/s0731-7085(03)00366-2 14656585

[10] Comission of the European Communities . 1996. Validation of analytical procedures. Proceedings of the International Conference on Harmonisation (ICH)

[11] Mendez A , Chagastelles P , Palma E , Nardi N , Schapoval E . 2008. Thermal and alkaline stability of meropenem: degradation products and cytotoxicity. Int J Pharm 350:95–102. doi:10.1016/j.ijpharm.2007.08.023 17904314

[12] Crandon JL , Ariano RE , Zelenitsky SA , Nicasio AM , Kuti JL , Nicolau DP . 2011. Optimization of meropenem dosage in the critically ill population based on renal function. Intensive Care Med 37:632–638. doi:10.1007/s00134-010-2105-0 21136037

[13] Sanford Guide . 2023. Sanford guide app version 6.1.5

[14] Grant EM , Zhong MK , Ambrose PG , Nicolau DP , Nightingale CH , Quintiliani R . 2000. Stability of meropenem in a portable infusion device in a cold pouch. Am J Health Syst Pharm 57:992–995. doi:10.1093/ajhp/57.10.992 10832500

[15] Manning L , Wright C , Ingram PR , Whitmore TJ , Heath CH , Manson I , Page-Sharp M , Salman S , Dyer J , Davis TME . 2014. Continuous infusions of meropenem in ambulatory care: clinical efficacy, safety and stability. PLoS One 9:e102023. doi:10.1371/journal.pone.0102023 25019523 PMC4096762

[16] Jamieson C , Allwood MC , Stonkute D , Wallace A , Wilkinson A-S , Hills T , BSAC Drug Stability Working Party . 2020. Investigation of meropenem stability after reconstitution: the influence of buffering and challenges to meet the NHS yellow cover document compliance for continuous infusions in an outpatient setting. Eur J Hosp Pharm 27:e53–e57. doi:10.1136/ejhpharm-2018-001699 32296506 PMC7147560

[17] Takasu Y , Yoshida M , Tange M , Asahara K , Uchida T . 2015. Prediction of the stability of meropenem in intravenous mixtures. Chem Pharm Bull (Tokyo) 63:248–254. doi:10.1248/cpb.c14-00516 25832020

[18] Tomasello C , Leggieri A , Cavalli R , Di Perri G , D’Avolio A . 2015. In vitro stability evaluation of different pharmaceutical products containing meropenem. Hosp Pharm 50:296–303. doi:10.1310/hpj5004-296 26448659 PMC4589882

